# Use of complementary and alternative therapies by patients with eye diseases: a hospital-based cross-sectional study from Palestine

**DOI:** 10.1186/s12906-020-03188-9

**Published:** 2021-01-04

**Authors:** Dania Jaber, Rafat Abu Ghannam, Waleed Rashed, Mohammad Shehadeh, Sa’ed H. Zyoud

**Affiliations:** 1 Department of Medicine, College of Medicine and Health Sciences, An-Najah National University, Nablus, 44839 Palestine; 2 Department of Ophthalmology, An-Najah National University Hospital, 44839 Nablus, Palestine; 3 Department of Clinical and Community Pharmacy, Department of Pharmacy, College of Medicine and Health Sciences, An-Najah National University, Nablus, 44839 Palestine; 4 Poison Control and Drug Information Center (PCDIC), College of Medicine and Health Sciences, An-Najah National University, Nablus, 44839 Palestine; 5 Clinical Research Center, An-Najah National University Hospital, Nablus, 44839 Palestine

**Keywords:** Alternative medicine, CAT, Eye diseases, Palestine

## Abstract

**Background:**

Generally, complementary and alternative therapies (CAT) are accepted methods of treatment by patients with various types of conditions. Their use is becoming especially prevalent among patients with eye problems even in developed countries. Thus, we aimed to determine the pattern of use of CAT in this patient population, to identify the patient characteristics associated with the use of CAT, and to assess the types of CAT used.

**Methods:**

A descriptive, cross-sectional study was conducted in Palestine at An-Najah National University Hospital between the time periods of October 2019 to May 2020, using questionnaire-based face to face interviews. Data were collected through convenience sampling. Patients responded to the questionnaire, which was focused on information adapted from previous research in this area, covering socio-demographic and clinical characteristics, types of CAT, source of information, and side effects on CAT use.

**Results:**

A total of 86 patients were interviewed for our study. Over two thirds, 67% reported using CAT for the specific purpose of improving their eye condition, and about one third (29.1%) received more than one therapy. The most common therapies reported were duea’ (i.e. supplication) (47.1%) and herbal therapies (24.1%). It was shown that patients with bilateral involvement of their eyes were almost twice more likely to describe using CAT than patients with unilateral eye pathology (*p* = 0.006). Also, patients who underwent surgery as their route of treatment were significantly less likely to use CAT (*p* = 0.043). Most of our study participants mentioned a non-physician source as their source of information regarding CAT with family members being the most frequently mentioned (30.2%) followed by the internet (25.6%) and friends (19.8%).

**Conclusions:**

The prevalence of CAT use among patients with eye disease is somewhat high in our study population. Because CAT may trigger adverse reactions, influence the progression of the disease, and interfere with conventional treatment, the ophthalmologist should frequently be asked patients with such diagnostics regarding the use of these therapies. Further work is required to analyze the mechanisms of action and to establish realistic guidelines for the use of these modalities.

**Supplementary Information:**

The online version contains supplementary material available at 10.1186/s12906-020-03188-9.

## Background

The use of complementary and alternative therapies (CAT) is on the rise throughout the world, particularly in the developed world [1]. CAT as a general term encompasses several specific medical and health systems, procedures, and products that are not commonly considered to be part of conventional medicine [1, 2]. However, the concept “alternative” refers to interventions that are used instead of conventional medical treatments, and the concept “complementary” refers to interventions that are used along with conventional medical treatments [3].

The use of CAT continues to increase; they are continuing to rise as an important element in our health care although it must be noted that some of these treatment alternatives have no proven clinical effects. Mostly ophthalmologists just use four key classes of eye acting medicines; anti-infective, anti-inflammatory, anti-glaucoma medications, and lubricants [4]. The eye has several natural mechanisms to defend itself against insults, when these mechanisms are disrupted ocular damage occurs. Conventional therapies are often used in treating eye disease, however, many limitations are faced from under-use to overuse and misuse leading to changes in the course of treatment [5]. Biologically based therapies (e.g. herbal, vitamins, and diet) and mind-body medicine (e.g. relaxation, praying, yoga, and breathing exercises) are the most commonly used.

Despite the widespread use of CAT at a global level in a wide variety of communities, especially in patients with eye diseases [6–14], there is inadequate data regarding the use of CAT in Palestine. To our understanding, several publications discussed the use of CAT and herbal therapies among Palestinians with diseases other than eye conditions, such as hypertensive patients [15], diabetes mellitus patients [16], coronary heart disease patients [17], hemodialysis patients [18], cancer patients [19], and the general public [20]. As for herbal therapies, there were some publications for its use among cancer patients [21], geriatric patients [22], and in pregnancy [23, 24]. Thus, we aimed to determine the pattern of use of CAT in this patient population, to identify the patient characteristics associated with the use of CAT, and to assess the types of CAT used. These findings should make an important contribution to this field since implementing CAT within the conventional medical practice in future healthcare systems relies, to a great extent, on the knowledge and attitude that patients have regarding CAT. In addition, this study aims to contribute to this growing area of research by the inclusion of CAT into medical curricula in Palestine has been accentuated by the more positive attitudes toward it, which have emerged from education on CAT.

## Methods

### Study design

A Cross-sectional study was conducted in Palestine among patients with eye diseases.

### Study area and population of the study

The study was conducted at the ophthalmic center at An-Najah National University Hospital (NNUH).

### Sample size and sampling methods

For the selection of the sample size, the convenience sampling method was used. The overall sample size for the study was 86 respondents.

### Inclusion criteria and exclusion criteria

All patients with eye disease who present at An-Najah ophthalmology outpatient clinics during the study timeframe with a diagnosis of eye disease were included in this study. Patients without a diagnosis of eye disease during the interview period were excluded.

### Data collection instruments

Structured face-to-face interviews were conducted at NNUH, over 5 months, to obtain information related to CAT, using a questionnaire based on previous studies about CAT inside and outside Palestine [8, 25]. The questionnaire was evaluated by specialists in ophthalmology, clinical pharmacy and toxicology, and biostatistics. Face-to-face interviews were chosen to include patients who have impaired vision and/or difficulty reading in our study. All data collected in the questionnaire were reported by the study respondents including their diagnosis of eye disease. A pilot study that included 10 patients was conducted first, to assess the clarity of the questionnaire. The questionnaire consisted of four sections (Additional file 1):

- The first section was about socio-demographic characteristics including age, gender, marital status, child-care giving, educational level, occupational status, income, residence, place of birth, health insurance, chronic co-morbidities, and lifestyle that is- smoking and exercise.
- The second section described the clinical characteristics of the participants including ophthalmologic diagnosis, symmetry and chronicity of eye disease, route of treatment, and ocular co-morbidities.
- The third section of the questionnaire focused on the regular use of CAT for eye diseases after the diagnosis of eye diseases. CAT types were subdivided into four main categories based on previous studies conducted in Palestine regarding CAT [15–18, 20, 26–30]: 1) Biologically based therapies being the first one, which included nutritional supplements, honey, diet, olive oil, warm and cold compressors and twenty- five different types of the most commonly used herbal remedies in Palestine. 2) Manipulative body-based therapies such as massage. 3) Mind-body medicine such as yoga and meditation. Islamic remedies such as duea’ (i.e. supplication), Ruqayya (i.e. faith healing), and Zamzam water were added to this category. 4) Alternative medical techniques such as homeopathy and reflexology. All CAT types included in this study were used for the specific purpose of improving eye disease.
- In the fourth section, participants were asked to provide a source of information and adverse effects immediately occurred after the use of a specific type of CAT.

### Ethical approval

All aspects of the study protocol were authorized by the Institutional Review Boards (IRB) (Protocol # AN7Nov2019) and the local health authorities before initiation of this study.

### Statistical analysis

Data were inserted and analyzed using the statistical package for social sciences problems version 15 (SPSS). Categorical variables were interpreted as frequencies (percentage) whereas continuous variables were interpreted as means ± SD or median [interquartile range]. The Chi-Square and the Fisher exact tests, as pertinent, were used for statistical significance testing between categorical variables. The Kolmogorov-Smirnov test was used to check the normality of the data. The Mann–Whitney U test was used to compare users and non-users of CAT in terms of age. The significance was set at *p*-value < 0.05.

## Results

### Socio-demographic data and clinical characteristics

A total of 86 patients were interviewed for our study at NNUH over a time period of 4 months. Ninety-one patients at NNUH qualified for our study inclusion criteria but eighty-six consented to be interviewed giving a response rate of 94.5%. All of the participants in our sample have the same religion and ethnicity (Islamic religious and Arabic ethnicity). Our study included 39 male- and 47 female participants (male to female ratio = 1:1.2) with ages ranging from 18 to 79 (mean = 43.58 ± 18.15 years). The majority of the study respondents were born in Palestine (95.3%) with 43 subjects residing in a city and 43 residing in a village. Ophthalmologic diagnoses were categorized according to the site of eye pathology. In addition to being in close proximity, in terms of the number of the patients diagnosed with them in our study, lens (*n* = 30) and retinal (*n* = 31) disease were the most common diagnoses (34.9%) and (36%) respectively. Whereas 14 (16.3%) patients suffered from corneal disease and 11 (12.8%) patients suffered from other pathologies including conjunctival and optic nerve disease. Approximately one third (31.4%) reported the presence of concurrent ocular comorbidity. Socio-demographic data and clinical characteristics are illustrated in Tables 1 and 2.

**Table 1:** Socio-demographic characteristics and their association with CAT use

Variable	Overall ***N*** = 86 (%) or median [interquartile range]	CAT users ***N*** = 58 (%) or median [interquartile range]	Non-CAT users ***N*** = 28 (%) or median [interquartile range]	***P-***value
**Age (year)**	42.5[25–60]	43.5[25–57]	42.5[25–60]	0.814 ^a^
**Age category (year)**				0.618 ^b^
18–39	38 (44.2)	27 (46.6)	11 (39.3)	
40–59	26 (30.2)	18 (31.0)	8 (28.6)	
≥ 60	22 (25.6)	13 (22.4)	9 (32.1)	
**Gender**				0.889 ^b^
Male	39 (45.3)	26 (44.8)	13 (46.4)	
Female	47 (54.7)	32 (55.2)	15 (53.6)	
**Marital status**				0.375 ^b^
Unmarried	27 (31.4)	20 (34.5)	7 (25.0)	
Married	59 (68.6)	38 (65.5)	21 (75.0)	
**Childcare giving**				0.645 ^b^
Yes	43 (50.0)	28 (48.3)	15 (53.6)	
No	43 (50.0)	30 (51.2)	13 (46.4)	
**Education level**				0.992 ^b^
Primary school	30 (34.9)	20 (34.5)	10 (35.7)	
High school	19 (22.1)	13 (22.4)	6 (21.4)	
University	37 (43.0)	25 (43.1)	12 (42.9)	
**Occupation**				0.284 ^b^
Employed	42 (48.8)	26 (44.8)	16 (57.1)	
Unemployed	44 (51.2)	32 (55.2)	12 (42.9)	
**Monthly Income**				0.357 ^b^
< 2000 NIS	43 (50.0)	27 (46.6)	16 (57.1)	
> 2000 NIS	43 (50.0)	31 (53.4)	12 (42.9)	
**Place of birth**				0.607 ^c^
Palestine	82 (95.3)	55 (94.8)	27 (96.4)	
Outside Palestine	4 (4.7)	3 (5.2)	1 (3.6)	
**Residence**				0.645 ^b^
Village	43 (50.0)	28 (48.3)	15 (53.6)	
City	43 (50.0)	30 (51.7)	13 (46.4)	
**Health insurance**				0.695 ^b^
Governmental	52 (60.5)	36 (62.1)	16 (57.1)	
Private	17 (19.8)	12 (20.7)	5 (17.9)	
No insurance	17 (19.8)	10 (17.2)	7 (25.0)	
**Chronic comorbid disease**				0.652 ^b^
Present	40 (46.5)	26 (44.8)	14 (50.0)	
Absent	46 (53.5)	32 (55.2)	14 (50.0)	
**Exercise**				0.282 ^b^
Never	27 (31.4)	15 (25.9)	12 (42.9)	
Sometimes	37 (43.0)	27 (46.6)	10 (35.7)	
Most of the time	22 (25.6)	16 (27.6)	6 (21.4)	
**Smoking**				0.944 ^b^
Smoker	25 (29.1)	17 (29.3)	8 (28.6)	
Non-smoker	61 (70.9)	41 (70.7)	20 (71.4)	

^a^ Statistical significance value calculated using the Mann–Whitney U test

^b^ Statistical significance value calculated using Chi-Square

^c^ Statistical significance value calculated using Fisher’s exact test

NIS = New Israeli Shekels (1 NIS = 0.29 US Dollars)

**Table 2:** Clinical characteristics and their association with CAT use

Variable	Overall N = 86 (%)	CAT users N = 58 (%)	Non-CAT users N = 28 (%)	***P-***value
**Diagnosis**				0.712 ^a^
Corneal disease	14 (16.3)	9 (15.5)	5 (17.9)	
Lens disease	30 (34.9)	22 (37.9)	8 (28.6)	
Retinal disease	31 (36.0)	21 (36.2)	10 (35.7)	
Others	11 (12.8)	6 (10.3)	5 (17.9)	
**Symmetry of eye disease**				**0.006**^a^
Unilateral	40 (46.5)	21 (36.2)	19 (67.9)	
Bilateral	46 (53.5)	37 (63.8)	9 (32.1)	
**Chronicity of disease**				0.135 ^a^
Acute	22 (25.6)	12 (20.7)	10 (35.7)	
Chronic	64 (74.4)	46 (79.3)	18 (64.3)	
**Route of treatment**				
Topical	67 (77.9)	43 (74.1)	24 (85.7)	0.277 ^b^
Systemic	9 (10.5)	7 (12.1)	2 (7.1)	0.712 ^b^
Intra-ocular injections	18 (20.9)	15 (25.9)	3 (10.7)	0.157 ^b^
Surgical	48 (55.8)	28 (48.3)	20 (71.4)	**0.043** ^a^
**Ocular co-morbidities**				0.695 ^a^
Absent	59 (68.6)	39 (67.2)	20 (71.4)	
Present	27 (31.4)	19 (32.8)	8 (28.6)	

^a^ Statistical significance value calculated using Chi-Square

^b^ Statistical significance value calculated using Fisher’s exact test

When asked about CAT, 58 (67.4%) patients reported using one or more types of CAT specifically for benefiting their eye diseases. Our study data showed no significant association between socio-demographics and CAT use (*p* > 0.05). It was shown, however, that patients with bilateral involvement of their eyes were almost twice more likely to describe using CAT than patients with unilateral eye pathology (*p* = 0.006). In addition, patients who underwent surgery as their route of treatment were significantly less likely to use CAT (*p* = 0.043). Other clinical characteristics including the site of pathology, chronicity, and ocular comorbidities were not associated with CAT use (*p* > 0.05). Socio-demographics and clinical characteristics and their association with CAT use are shown in Tables 1 and 2.

### Types of CAT therapy used by the study participants

Among CAT users, 33 (38.4%) patients described using one type of CAT only while 25 (29.1%) patients reported using two or more. More than half of the study respondents (52.9%) reported using mind-body medicine therapies with duea’ (i.e. supplication) (47.1%) being the most frequently used, followed by Zamzam water (16.1%) and Ruqayya (i.e. faith healing) (14.9%). Biologically based therapies were used by 37 patients (42.5%), of which, herbal remedies were the most frequently used (24.1%), followed by diet (16.1%), cold compressors (14.9%), and nutritional supplements (11.5%). Of note, 5 out of 10 patients who used nutritional supplements reported using Omega 3. Table 3 shows the types of CAT and their frequency of use.

**Table 3:** Types of CAT reported by the study participants

CAT Type	Frequency (%)
**Biologically Based Therapies**	**37 (42.5)**
Herbal therapies	21 (24.1)
Diet	14 (16.1)
Cold compress	13 (14.9)
Nutritional supplements	10 (11.5)
Warm compress	7 (8.0)
Olive oil	5 (5.7)
Honey	4 (4.6)
**Manipulative Body-Based Methods**	**11 (12.6)**
Massage therapy	10 (11.5)
Physiotherapy	2 (2.3)
**Mind-Body Medicine**	**46 (52.9)**
duea’ (i.e. supplication)	41 (47.1)
Zamzam water	14 (16.1)
Ruqayya (i.e. faith healing)	13 (14.9)
Aerobic exercise	12 (16.1)
Relaxation techniques	8 (9.2)
Walking	7 (8.0)
Breathing exercise	7 (8.0)
Music	4 (4.6)
Cupping	2 (2.3)
Meditation	2 (2.3)
Yoga	1 (1.1)
Islamic Nasheed	1 (1.1)
Isolation	1(1.1)
**Alternative Medical System**	**3 (3.4)**
Homeopathy	2 (2.3)
Reflexology	1 (1.1)

### Types of herbs

Fourteen different herbal remedies were used, with *CATellia Sinensis* being most frequently reported (18.4%) followed by *Matricaria chamomilla* (10.3%) and *Mentha spicata* (5.5%). Ten patients reported using one herbal remedy (11.6%), six (7.0%) reported using 2 herbal remedies, and five (5.8%) reported using more than 3 herbal remedies for their eye disease. Table 4 shows the distribution of the use of herbal therapies.

**Table 4:** Distribution of herbal therapy use among study participants

Herbs	Frequency (%)
Tea leaves *(Camellia sinensis)*	16 (18.4)
Chamomile *(Matricaria chamomilla)*	9 (10.3)
Mint (*Mentha spicata)*	5 (5.5)
Anise *(Pimpinella anisum)*	4 (4.6)
Common Sage *(Salvia officinalis)*	3 (3.4)
Fenugreek *(Trigonella Foenum graecum)*	3 (3.4)
Cumin *(Nigella sativa)*	2 (2.3)
Garlic *(Allium sativum)*	2 (2.3)
Lemon *(Citrus limon)*	2 (2.3)
Onion *(Allium cepa)*	2 (2.3)
Thyme *(Thymus vulgaris)*	2 (2.3)
*Aloe Vera (Aloe barbadensis)*	1 (1.1)
Fennel flower *(Foeniculum vulgare)*	1 (1.1)
Olive leaves *(Olea europaea)*	1 (1.1)

### Source of information regarding CAT use

Whilst twenty-six (30.2%) of our study respondents mentioned family members as their source of information, 25.6% mentioned the internet, and 19.8% mentioned friends as their source of information regarding CAT use. Table 5 shows the distribution of sources of information among study participants.

**Table 5:** Distribution of sources of information regarding CAT use among study participants

Source of information	Frequency (%)
Family members	26 (30.2)
The internet	22 (25.6)
Friends	17 (19.8)
Religious books	13 (15.1)
Doctors	11 (12.1)
Social media	9 (10.5)
Television	9 (10.5)
Places of worship	5 (5.8)
Scientific magazines	5 (5.8)
Pharmacists	3 (3.5)
Scientific books	3 (3.5)
University	2 (2.3)
School	1 (1.2)

### Patient-reported adverse effects associated with CAT use

It is noteworthy to mention that 11 different adverse effects, attributed to using a CAT, were described by our study respondents. Vertigo was the most frequently mentioned (4.7%) followed by fatigue, abdominal pain, concentration difficulties, and headache (3.5%). Other adverse effects include thirst, nausea, insomnia, and bad taste as shown in Table 6.

**Table 6:** distribution of adverse effects of CAT among study participants

Adverse effects	Frequency (%)
Vertigo	4 (4.7)
Fatigue	3 (3.5)
Abdominal pain	3 (3.5)
Concentration difficulties	3 (3.5)
Headache	3 (3.5)
Thirst	2 (2.3)
Nausea	2 (2.3)
Insomnia	2 (2.3)
Bad taste	2 (2.3)
Skin rash	1 (1.2)
Vomiting	1 (1.2)

## Discussion

In this cross-sectional study, we observed the patterns of CAT use in a population of patients with various types of eye diseases, attending ophthalmology outpatient clinics at NNUH, over 4 months. Many other studies have discussed the use of CAT among patients with specific eye diseases, such as glaucoma and inflammatory eye conditions [8, 12]. Both studies gathered data on certain therapies that were specifically thought to improve eye disease using similar definitions of CAT. In contrast to the previously mentioned studies, this study included all patients with eye conditions regardless of the diagnosis in addition to being the first one to assess CAT use for eye diseases in Palestine.

Within our sample, over two-thirds reported using CAT with the particular intention of improving their eye conditions, and approximately one third confirmed the use of more than one type of therapy. While a broad variety of CAT has been mentioned by our patients, the most commonly reported therapies were duea’ (i.e. supplication), herbal therapies, Zamzam water, and diet. Taking into consideration that previously conducted studies regarding CAT use in Palestine, confirmed that herbal remedies use was quite prevalent [18, 22–24], we investigated the use of 25 different types of herbs. Not unexpectedly, *Camellia sinensis* and *Matricaria chamomilla*, which are readily available and inexpensive, were the most frequently used. Nevertheless, patients who underwent surgical management as their route of treatment were significantly less likely to use CAT compared with those who didn’t have surgery whereas patients who had bilateral eye involvement, were significantly more likely to use CAT compared to patients suffering from unilateral eye disease.

Consistent with the literature, this research found that 11% of parents reported prior CAT use for their child’s eye condition, and 44% of parents, depending on the side effects, reported a preference for CAT use for their child’s eye condition [31]. Furthermore, in another previous study, the total prevalence of CAT use for glaucoma was around 5% of the population of patients already receiving traditional treatment [13]. Also, approximately 14% of glaucoma patients in another cross-sectional study reported current or past use of CAT for their glaucoma [14]. The use of CAT therapy for inflammatory eye disease tends to be more popular (42%) as shown in Smith et al. study [8].

In accordance with previously published data [8, 18], when asked about the source of information regarding CAT use, family and friends were amongst the most frequently described, bearing in mind that only 12.1% mentioned their physician as their source of information. As for the side effects, they were relatively mild and uncommon, with vertigo being the most frequently reported by 4.7% followed by fatigue, abdominal pain, and concentration difficulties. Vomiting and skin rash were the least reported ones.

It can be concluded that specific CAT modalities are on the rise in our population. Notwithstanding that some of these alternative remedies have been shown to have beneficial effects concerning eye disease such as, tea tree oil which was found to have some antiviral activity against HSV-1 and HSV-2 [32, 33] and honey which was shown to fasten corneal epithelium healing time [32, 34]. Several studies have shown the great value of CAT use and natural remedies, there has been an increase in their use globally due to their perceived effectiveness and low cost relative to conventional therapies [32, 35–38]. It could reduce the usage of conventional methods especially antimicrobials thus limiting the creation of new strains of resistive microbes. Furthermore, honey has been used for centuries as a natural remedy, which helps in healing of wounds and provides an antibacterial effect, the most widely known is a type developed in New Zealand known as Manuka honey which is believed to have an antibacterial effect on 60 different species [32]. Another well-known natural remedy that was used in previous centuries is a member of the Liliaceae family called *Aloe Vera* which was used by many healthcare facilities in a variety of clinical conditions from wound care to diabetes [32]. On the other hand, CAT may affect the outcome of disease, cause adverse effects, and interact with conventional treatment [8, 39]. So, further research is needed to improve our understanding of CAT and to evaluate their usefulness and utility for ophthalmologic purposes [7].

The reasoning behind the use of nutritional supplements in glaucoma is backed by a substantial amount of literature showing that natural anti-inflammatory, antioxidant and anti-apoptotic compounds are effective in preventing the death of retinal ganglion cells in in vitro and in vivo retinal degeneration models [40–43]. In a comprehensive systematic review of the use of CAT in the care of ophthalmology patients and their possible therapeutic benefits, Welte et al. [44] found that phytotherapeutic approaches have produced significant results in half of the randomized trials published, while acupuncture or acupressure have yielded few significant benefits. Many of the widely used CAT have no clinical evidence to support them or have been closely connected with complications and non-compliance with medical treatment [25, 45]. Either way, healthcare providers are encouraged to become familiar with the most commonly used CAT so they can inform their patients about the possible benefits and risks of using such therapies [8].

## Strengths and limitations

The main strengths of this study are that: it takes into account multiple groups of eye diseases, and it is the first to assess CAT use among patients with eye diseases in Palestine. However, the following limitations should be underlined. First, the cross-sectional design of the study and its small sample size. Second, self-reported data by our patients which might be prone to recall or desirability bias, as well as the convenience sampling method that should be taken into consideration as another source of bias. An additional limitation that should be reported is the potential bias introduced by the researcher helping respondents complete the questionnaire. Fourth, this study did not assess physicians or other healthcare providers for their attitudes and practices toward CAT. Another issue is the absence of a control group in our study to compare the habits of CAT in individuals without eye disease in order to evaluate any specific pattern for eye diseases.

## Conclusions

To conclude, the prevalence of CAT use among patients with eye disease is somewhat high in our study population. In the light that the tendency toward CAT use may not be associated with any particular socio-demographic or clinical characteristics, physicians need to be aware that their patients might have used or might be using CAT and need to address their possible risks and benefits. It is worth mentioning that, there are limited data regarding CAT in eye diseases, further research is needed to evaluate the mechanisms of action and to develop practical guidelines for the use of these modalities. In addition, further research is required to establish the therapeutic efficiency of CAT by providing the visual acuity information of the patients before and after the usage of CAT in order to understand whether the use of these therapies helps the regular treatment for eye diseases. Because CAT can cause adverse effects, affect the course of the disease, and interfere with conventional therapy, ophthalmologists will regularly question patients with these diagnoses about the use of such therapies. Nevertheless, due to patient interest and the possible increase in the usage of alternative and complementary approaches, further work is clearly justified in developing consistent recommendations and clear guidelines for intervention for such treatments.

## Supplementary Information

**Additional file 1 Study questionnaires.** This is the final version of the English version that was used to obtain data that will help to examine the use of complementary and alternative therapies (CAT) among patients for treating eye disease, the reasons and factors influencing their use, and the types of CAT used.

## Data Availability

The datasets used and/or analyzed during the current study are available from the corresponding author on reasonable request.

## Abbreviations

CAT: Complementary and Alternative Therapies
IRB: Institutional Review Board
NNUH: An-Najah National University Hospital.
