# Supplementary material for: Use of complementary and alternative therapies by patients with eye diseases: a hospital-based cross-sectional study from Palestine
Source: BMC Complement Med Ther. 2021 Jan 4;21:3. doi: 10.1186/s12906-020-03188-9 (PMC7784325; doi:10.1186/s12906-020-03188-9)
Supplement: Supplementary file 1 — Additional file 1 Study questionnaires. This is the final version of the English version that was used to obtain data that will help to examine the use of complementary and alternative therapies (CAT) among patients for treating eye disease, the reasons and factors influencing their use, and the types of CAT used. [file 12906_2020_3188_MOESM1_ESM.doc]

**Additional file 1: Study questionnaires. This is the final version of the English version that was used to obtain data which will help to examine the use of complementary and alternative therapies (CAT) among patients for treating eye disease, the reasons and factors influencing their use, and the types of CAT used.**

**Section one: personal information:**

| **Age:** | **……………………………….** |  |  |  |
| --- | --- | --- | --- | --- |
| **Gender:** | **Male** | **Female** |  |  |
| **Marital status:** | **Single** | **Married** |  |  |
| **Child caregiver:** | **Yes** | **No** |  |  |
| **Education:** | **Non-high school graduate** | **High school graduate** | **University graduate** |  |
| **Employment:** | **Employed** | **Unemployed** |  |  |
| **Income:** | **Less than 2000** | **2000-5000** | **5000-10000** | **More than 10000** |
| **Place of birth:** | **Palestine** | **Other** |  |  |
| **Residence:** | **Village** | **City** | **Refugee camp** |  |
| **Health insurance:** | **Governmental** | **Private** | **None** |  |
| **Chronic co-morbid disease:** | **Present** | **Absent** |  |  |
| **Lifestyle:** |  |  |  |  |
| How often do you exercise? | Never | Sometimes | Most of the time | Always |
| Are you a smoker? | Yes | No |  |  |

**Section two: clinical information:**

| **Diagnosis:** | **………………………………………** | | | |  |  |  |
| --- | --- | --- | --- | --- | --- | --- | --- |
| **Symmetry:** | **Unilateral** | | | | **Bilateral** |  |  |
| **In case of eye inflammation:** |  | | | |  |  |  |
| Site: | Eyelid | | | | Conjunctiva | Lacrimation ducts |  |
|  | Cornea | | | | Iris | Vitreous humor |  |
|  | Retina | | | |  |  |  |
| Duration: | Acute | | | | Recurrent acute | Chronic |  |
| **Visual acuity:** | **0-0.3** | | | | **0.4-0.9** | **More than 1** |  |
| **Duration:** |  | | | |  |  |  |
| **Route of treatment:** | **Systemic** | **Oral** | **IM** | **IV** | **Topical** | **Intra-ocular** | **Surgical** |
| **Other eye diseases:** | **………………………………………** | | | |  |  |  |

**Section 3: Use of complementary and alternative therapies (CAT) in eye related diseases:**

- **Biologically based therapies:**

| **Vitamins:** | **A** | **B** | **C** | **D** | **E** | **K** | **Omega 3** |
| --- | --- | --- | --- | --- | --- | --- | --- |
| **Honey** | **Yes** | | **No** | |  | |  |
| **Diet:** | **yes** | | **No** | |  | |  |
| **Olive oil:** | **yes** | | **No** | |  | |  |
| **Cold compress:** | **yes** | | **No** | |  | |  |
| **Warm compress:** | **yes** | | **No** | |  | |  |
| **Herbal therapies** | **Common sage** | | **Mint** | | **Cumin** | | **Mastic** |
|  | **Tea leaves** | | **Thyme** | | **Caraway** | | **Cinnamon** |
|  | **Vervain** | | **Chamomile** | | **Anise** | | **Parsley** |
|  | **Garlic** | | **Fenugreek** | | **Olive Leaves** | | **Senna** |
|  | **Hawthorn** | | **Ginger** | | **Ginseng** | | **Pricky pear** |
|  | **Lemon** | | **Fennel flower** | | **Onion** | | **Liquorice** |
|  | **Belladonna** | |  | |  | |  |
|  |  | |  | |  | |  |

- **Manipulative body- based methods:**

| **Massage therapy** | **yes** | **No** |
| --- | --- | --- |
| **Physiotherapy** | **yes** | **No** |

- **Mind-Body Medicine:**

| **Ruqayya (faith healing)** | **Breathing exercise** | **Cupping** | **Relaxation techniques** | |
| --- | --- | --- | --- | --- |
| **Exorcism** | **Meditation** | **Walking** | **Hypnosis** | |
| **Music** | **Islamic nashid** | **Isolation** | **Yoga** | |
| **Zamzam water** | **Aerobics** | **Duea’(supplication)** | |  |

- **Alternative medical systems:**

| **Chinese acupuncture** | **yes** | **No** |
| --- | --- | --- |
| **Reflexology** | **yes** | **No** |
| **Homeopathy** | **yes** | **No** |

- **Source of information:**

| **Doctor** | **Family members** | **Pharmacist** | **Social media** |
| --- | --- | --- | --- |
| **TV** | **Worship places** | **Friends** | **Internet** |
| **Scientific magazines** | **Religious books** | **Scientific books** | **School** |
| **Universities** | **Others:** |  | |

- **Side effects:**

| **Thirst** | **Nausea** | **Vertigo** | **Fatigue** |
| --- | --- | --- | --- |
| **Abdominal pain** | **Difficulty concentrating** | **Insomnia** | **Headache** |
| **Rash** | **Constipation** | **Diarrhea** | **Vomiting** |
| **Bad taste** | **Others:** |  | |

- **Cost of Alternative Medicine:**

| **Monthly Cost** | **Less than 200 NIS** | **From 200-500 NIS** | **More than 500 NIS** |
| --- | --- | --- | --- |
